# Doença de Fabry e seus Diferentes Fenótipos

**DOI:** 10.36660/abc.20240535

**Published:** 2025-03-18

**Authors:** Murillo Oliveira Antunes, Rafael Ruas Nastari, Edmundo Arteaga-Fernandez, Marcelle G. Henriques Lizandro, William Batah El-Feghaly, Guilherme José dos Santos Ferreira, Alan Silva Martins, Juliana Alzira Gonzales Oliveira Leguizamon, Vinicius Machado Correia, Vagner Madrini, Fábio Fernandes

**Affiliations:** 1 Hospital das Clínicas Faculdade de Medicina Universidade de São Paulo São Paulo SP Brasil Instituto do Coração do Hospital das Clínicas da Faculdade de Medicina da Universidade de São Paulo, São Paulo, SP – Brasil; 2 Universidade São Francisco Bragança Paulista SP Brasil Universidade São Francisco, Bragança Paulista, SP – Brasil

**Keywords:** Doença de Fabry, Cardiomiopatias, Genética

## Abstract

A Doença de Fabry (DF) é uma condição genética ligada ao cromossomo X, causada por variantes no gene *GLA* , que resultam na deficiência da enzima α-galactosidase A e no acúmulo de globotriaosilceramida (Gb3) em tecidos como o coração, rins e sistema nervoso. Este estudo relata uma série de casos envolvendo pacientes diagnosticados com DF, destacando a diversidade fenotípica da doença, que pode ser confundida com outras condições cardiológicas. A avaliação genética, aliada à dosagem de biomarcadores e à atividade enzimática da α-galactosidase, quando bem indicadas, é essencial para o diagnóstico preciso. O diagnóstico precoce da DF é fundamental para a implementação de tratamentos que retardem a sua progressão, além de evitar complicações graves, reforçando a necessidade de maior conscientização entre os cardiologistas sobre essa condição.

## Introdução

A Doença de Fabry (DF) é uma desordem lisossômica de armazenamento ligada ao cromossomo X, causada por variantes patogênicas no gene *GLA* , que resultam em uma redução significativa da atividade da enzima α-galactosidase A (α-GalA). Isso leva ao acúmulo de globotriaosilceramida (Gb3) em tecidos como coração, rins, vasos sanguíneos e sistema nervoso periférico. Embora as estimativas de incidência variem, estudos recentes, incluindo triagens neonatais e análises de biobanco sugerem que a prevalência real pode ser subestimada, com acometimento de até 1 em 5.732 para variantes de início tardio.^1^ Com a introdução da terapia de reposição enzimática (TRE), o diagnóstico precoce tornou-se crucial para retardar a progressão da doença. Esta série de casos visa demonstrar as diferentes apresentações fenotípicas da DF, que podem mimetizar outras cardiopatias, facilitando assim o reconhecimento precoce da condição.

### Caso 01 – Cardiomiopatia hipertrófica apical

**Figura 1:**
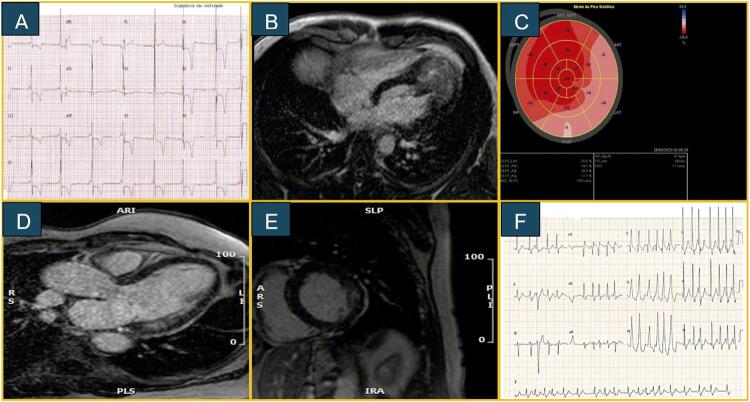
– Caso 1: (A) Eletrocardiograma com intervalo PR curto e inversão de onda T de V3 a V6. (B) RMC demonstrando espessamento apical do VE. Caso 2: (C) Ecocardiograma com strain demonstrando acometimento do VE em regiões anteriores, lateral e inferolateral basais. RMC com realce tardio na parede lateral do VE em eixo longo (D) e eixo curto (E). Caso 3: (F) Eletrocardiograma com fibrilação atrial e bloqueio de ramo direito.

Paciente masculino, 29 anos, sem comorbidades ou história familiar de cardiopatia, queixava-se de formigamento e queimação nas pernas, assim como uma mancha em região abdominal (angioqueratomas). Procurou um serviço de saúde devido precordialgia atípica ( Tabela 1 ). O eletrocardiograma (ECG) mostrou intervalo PR curto e sinais de sobrecarga ventricular esquerda (SVE) com inversão de ondas T em V3-V6 ( Figura 1 ). O ecocardiograma revelou hipertrofia assimétrica do ventrículo esquerdo (VE) na região apical, com espessura máxima de 17 mm e ausência de obstrução na via de saída do VE, sendo inicialmente diagnosticado com cardiomiopatia hipertrófica (CMH) não obstrutiva, forma apical. A ressonância magnética cardíaca (RMC) evidenciou hipertrofia circunferencial nos segmentos médios e apicais, com espessura de 21 mm e realce tardio miocárdico multifocal de padrão não isquêmico ( Figura 1 ). A investigação genética revelou uma variante patogênica no gene *GLA* ( Tabela 2 ), e a dosagem da enzima alfa-galactosidase mostrou valor reduzido, confirmando o diagnóstico de DF. Foi realizado um rastreio familiar e encontrado a mesma variante na mãe do paciente.

**Tabela 1:** – Características clínico-laboratoriais

	Caso #1	Caso #2	Caso #3	Caso #4	Caso #5	Caso #6
Idade, anos	29	57	52	40	68	64
Sexo	Masculino	Feminino	Feminino	Masculino	Feminino	Masculino
Fenótipo	CMH apical	Amiloidose cardíaca	Fibrilação atrial	Assintomático	(Insuficiência Cardíaca com Fração de ejeção preservada) ICFEp	(Insuficiência Cardíaca com Fração de ejeção reduzida) ICFEr
Apresentação clínica	Sintomas cardiovasculares – Dor precordial	Hipoidrose, Acroparestesia e palpitações	Palpitações, Acroparestesia e vertigem	Anidrose e intolerância ao calor	Sintomas cardiovasculares – Dispneia	Sintomas cardiovasculares – Dispneia
Intervalo PR curto	Sim	*	Sim	Sim	Sim	*
FEVE, %	Preservada	Preservada	Preservada	Preservada	Preservada	Reduzida
Espessura máxima, mm	21	15	21	12	19	17
Dosagem de α-galactosidase	Reduzido	Reduzido	-	Reduzida	-	Reduzida
Dosagem lyso-gb3 (VR até 0,8 ng/ml)	3,1	15,71	-	71,7	23,1	-
Comprometimento Renal	Não	Não	Sim	Sim	Não	Sim

* Ritmo cardíaco não sinusal. Fonte: Autoria própria.

**Tabela 2:** – Testes Genéticos

	Caso #1	Caso #2	Caso #3	Caso #4	Caso #5	Caso #6
Teste Genético	Painel	Sanger	Sanger	Sanger	Sanger	Painel
Sequência referência	NM_000169.3	NM_000169.3	NM_00169.3	NM_00169.3	NM_00169.3	NM_00169.3
Coordenada Cromossômica (GRCh37)	chrX-100653021	chrX-100662818	chrX-100656700	chrX-100655733-100655734	chrX-100655733-100655734	chrX: 100653930
Variante c.DNA	c.1066C>T	c.73delG	c.467C>A	c.559_560del	c.559_560del	c.644A>G
Variante Proteína	p.Arg356Trp	p.Asp25Thf*96	p.Ala156Asp	p.Met187ValfsTer6	p.Met187ValfsTer6	p.Asn215Ser
Zigosidade	Hemizigose	Heterozigose	Heterozigose	Hemizigose	Heterozigose	Hemizigose
Classificação ACMG	Patogênica	Provavelmente Patogênica	Provavelmente Patogênica	Patogênica	Patogênica	Patogênica

Fonte: Autoria própria.

### Caso 02 – Fenótipo de amiloidose cardíaca

Paciente feminina, 57 anos, referia hipoacusia e zumbido de longa data assim como hipohidrose e dores em membros inferiores que se intensificavam com o frio durante a infância ( Tabela 1 ). Diagnóstico prévio de fibrilação atrial (FA) permanente e palpitações recorrentes, havia passado por cinco procedimentos de ablação sem sucesso. Seu histórico familiar era frusto para doenças de origem genética. O ecocardiograma mostrou hipertrofia concêntrica moderada do VE, com índice de massa de 114 g/m^2^ e fração de ejeção preservada. O strain longitudinal global (SLG) do VE era reduzido, principalmente nos segmentos basais das paredes anterior e lateral. A RMC revelou hipertrofia assimétrica do VE, predominando na região inferolateral, com espessura de 15 mm, realce tardio heterogêneo de padrão não isquêmico e T1 nativo miocárdico reduzido (852 ms, referência ~1000 ms) ( Figura 1 ). O teste genético identificou uma variante patogênica no gene *GLA* ( Tabela 2 ). A atividade da enzima alfa-galactosidase dosada foi reduzida, confirmando o diagnóstico de DF. A paciente tinha duas filhas e um filho, sendo que este último apresentou resultado positivo para a mesma variante no rastreio familiar.

### Caso 03 – Fibrilação atrial paroxística e insuficiência renal

Paciente feminina, 52 anos, em hemodiálise devido a doença renal crônica hipertensiva, foi encaminhada para avaliação cardiológica após um episódio de FA, revertido com amiodarona, durante uma das sessões de hemodiálise. Sempre referiu dores neuropáticas nas extremidades, que se intensificavam com frio e calor, assim como episódios frequentes de vertigem ( Tabela 1 ). Seu ECG mostrava ritmo sinusal e intervalo PR curto assim como bloqueio do ramo direito ( Figura 1 ). Foi solicitado um ecocardiograma que evidenciou espessura do septo interventricular de 21 mm e da parede lateral de 14 mm, com fração de ejeção preservada e sem obstrução na via de saída do VE.

A paciente apresentou um histórico familiar relevante, incluindo o falecimento da mãe por doença renal crônica, dois irmãos em hemodiálise e uma irmã com doença renal crônica não dialítica. Diante do histórico familiar associado a problemas renais e cardíacos, foi realizada uma análise genética que revelou uma variante patogênica no gene *GLA* ( Tabela 2 ), também constatada em seus irmãos, confirmando o diagnóstico de DF na família.

### Caso 04 – Assintomático com hipertrofia de parede lateral

**Figura 2:**
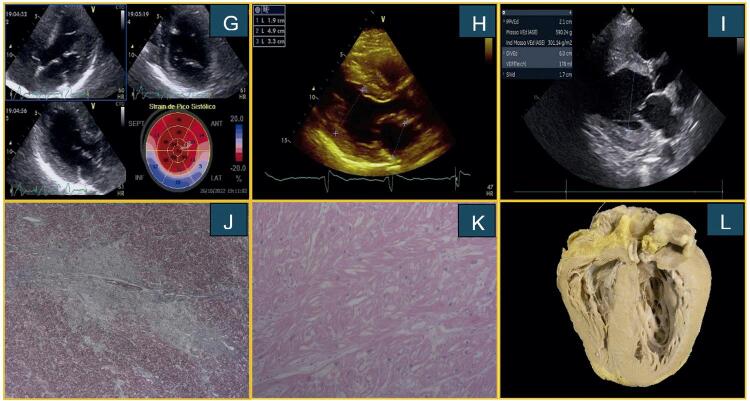
– Caso 4: (G) Imagens ecocardiográficas demonstrando espessamento do músculo papilar, aumento da espessura global do VE e técnica de Strain com acometimento do segmento basal das paredes inferior, lateral e infero-lateral. Caso 5: (H) Imagem ecocardiográfica, exibe espessamento da parede septal e lateral do VE. Caso 6: (I) Ecocardiograma revela espessamento do VE assim como diâmetro diastólico final aumentado. Imagens de microoscopia óptica, com coloração com tricrômico de Masson mostrando áreas de fibrose (J) e em coloração hematoxilina eosina demonstrando extensa vacuolização do citoplasma dos cardiomiócitos (K). (L) Vista coronal de coração explantado com aumento de espessura de todo miocárdio.

Paciente masculino, 40 anos, relatou intolerância ao calor e ausência de suor desde a infância ( Tabela 1 ). Referia que a mãe possuía o diagnóstico de arritmia cardíaca. Seu ECG mostrou ritmo sinusal com intervalo PR curto e sinais de SVE. No ecocardiograma, o índice de massa do VE foi de 142 g/m^2^, com espessura septal de 10 mm, parede lateral de 12 mm e hipertrofia dos músculos papilares ( Figura 2 ). Exames bioquímicos revelaram comprometimento da função renal e proteinúria de 1,55 g/24h. Diante desses achados, suspeitou-se de DF. A dosagem da atividade da enzima alfa-galactosidase mostrou-se reduzida ( Tabela 1 ) e o teste genético (Sanger) revelou uma variante patogênica no gene *GLA* ( Tabela 2 ), identificada de igual maneira em sua genitora.

### Caso 05 – Insuficiência cardíaca com fração de ejeção preservada

Paciente feminina, 68 anos, relatava dispneia aos esforços, que comprometiam significativamente suas atividades cotidianas. Referia episódios de dor precordial atípica, sem relação com esforço, ( Tabela 1 ) negando demais queixas. Questionada sobre seu histórico familiar, relatou que seu pai faleceu aos 45 anos devido a uma causa indeterminada. O ECG evidenciou intervalo PR curto, distúrbio de condução intraventricular e alterações difusas da repolarização ventricular. O ecocardiograma mostrou hipertrofia ventricular concêntrica do VE, assim como aumento das espessuras do septo interventricular e da parede lateral respectivamente de 19 mm e 18 mm. A massa indexada do VE calculada foi de 320 g/m^2^. Realizado SLG com redução de seus valores na região ínfero-latero-basal ( Figura 2 ). Um painel genético para avaliação de genocópias de CMH demostrou presença de variante patogênica no gene *GLA* , confirmando a DF ( Tabela 2 ). Dois filhos da paciente foram submetidos a rastreamento familiar e a variante foi identificada em um deles.

### Caso 06 – Insuficiência cardíaca com fração de ejeção reduzida

Paciente masculino, 64 anos, com histórico de CMH diagnosticada há 30 anos, sem outros sintomas sistêmicos ( Tabela 1 ), foi internado por descompensação de insuficiência cardíaca perfil C. O histórico familiar incluía um irmão também com o diagnóstico de CMH. Apesar do tratamento otimizado e da terapia de ressincronização cardíaca realizada três anos antes, o ecocardiograma revelou fração de ejeção do VE de 29% e hipertrofia ventricular com espessuras de septo e parede lateral de 17 mm ( Figura 2 ). Devido à refratariedade à retirada do inotrópico, o paciente foi considerado em fase terminal da doença e submetido com sucesso a transplante cardíaco.

A análise histopatológica da peça explantada ( Figura 2 ) e um painel genético ( Tabela 2 ) revelaram uma variante patogênica no gene *GLA* , com níveis reduzidos da atividade da enzima alfa-galactosidase. Seu irmão também foi sequenciado para o mesmo gene, sendo encontrada a mesma variante, de origem materna.

## Discussão

A DF é uma condição genética ligada ao cromossomo X que se manifesta de maneira diferente em homens e mulheres. Existem dois fenótipos principais: o clássico, mais comum em homens, caracterizado pela perda total da função da enzima α-GalA, resultando em sintomas multissistêmicos; e o não clássico, com variantes de *late-onset* que levam a uma progressão mais lenta da doença, geralmente afetando apenas o coração ou os rins.^2 , 3^ Novos estudos mostraram a tendência de variantes com perda de função gerarem um fenótipo com atividade enzimática bastante diminuída e quadro clínico clássico, enquanto variantes *missense* estão associadas a fenótipos de início tardio ( *late-onset* ).^4^ Na série descrita, três pacientes tinham variantes que resultaram em *stop codon* precoce, porém apenas o caso 4 evoluiu conforme descrito na literatura.

Não só a variante genética, mas o lyso-Gb3, forma desacetilada do Gb3, é um marcador da DF fortemente relacionado com seu fenótipo. Seu valor tende a ser elevado em pacientes com a forma clássica e mais baixo na forma não clássica.^5^

Pacientes do sexo masculino com DF de início tardio apresentam maior atividade residual de α-Gal A em comparação com a DF clássica, embora ainda muito abaixo dos valores normais.^1^ Em mulheres heterozigotas, a atividade de α-Gal A pode ser normal ou ligeiramente deficiente, muitas vezes com evolução clínica assintomática, o que desencoraja a dosagem sérica desse marcador nessa população, sendo o diagnóstico realizado por meio da confirmação do genótipo. Existe a possibilidade, em mulheres heterozigotas, da inativação do cromossomo X saudável, sendo o que contém a variante o que prepondera, gerando sintomas graves com a apresentação clínica clássica da doença. Essa expressão genética se dá de forma randômica em cada célula do organismo.^6^

Manifestações típicas da DF, como angioqueratomas e córnea verticilata, são menos frequentes, sendo mais comuns em fenótipos clássicos. Pacientes adultos com alterações estruturais no miocárdio, aumento progressivo ao longo do tempo do espessamento miocárdico, distúrbios de condução, doença renal crônica e sintomas neurológicos, especialmente acroparestesias e/ou hipohidrose devem ser investigados ativamente para DF, assim como seus familiares.

A manifestação cardíaca típica da DF é a hipertrofia ventricular esquerda concêntrica, comum a partir da terceira década de vida. Essa hipertrofia pode ser confundida com outras condições, como CMH e amiloidose cardíaca.^7^ Estudos indicam que a DF está presente em 0,5 a 3% dos casos diagnosticados como CMH.^8^ Na série de casos apresentada, dois pacientes tiveram o diagnóstico de DF erroneamente atribuído à CMH, resultando em atraso na TRE e, em um caso, a necessidade de transplante cardíaco.

No ECG, alterações como a redução do intervalo PR e a prevalência aumentada de FA são indicativas de DF. A ecocardiografia é essencial para o diagnóstico e monitoramento da cardiomiopatia de Fabry, revelando, entre outros achados, o espessamento da parede ventricular e a disfunção diastólica.^2^

A RMC também é útil, destacando a fibrose nos segmentos basais inferolaterais e a redução dos valores no mapa T1, que podem ser indicadores precoces da DF.

O tratamento específico consiste na TRE, que visa substituir a enzima que está em falta, evitando, consequentemente, o acúmulo de Gb3. No Brasil, temos a alfa-galsidase e a beta-galsidase, ambas administradas por via intravenosa a cada duas semanas. Mais recentemente, uma nova proposta de terapia, as chaperonas, surgiu para aumentar o arsenal terapêutico no tratamento da DF. Todavia, essa classe demonstrou eficácia apenas em subtipos específicos de variantes patogênicas, sendo necessária uma validação *in vitro* da medicação para a variante específica do paciente antes de sua aplicação. As chaperonas atuam mantendo a estabilidade da proteína disfuncional, auxiliando em seu correto dobramento e, consequentemente, preservando sua atividade.^9^

Por fim, uma investigação clínica detalhada, junto ao auxílio de uma avaliação genética conduzida por um profissional experiente, é crucial para o diagnóstico da DF. A capacitação constante dos cardiologistas é fundamental para aumentar o reconhecimento da doença e viabilizar intervenções precoces, que podem retardar sua progressão e prevenir complicações graves, garantindo um melhor prognóstico para os pacientes.
