# Supplementary material for: Doença de Fabry e seus Diferentes Fenótipos
Source: Arq Bras Cardiol. 2025 Mar 18;122(2):e20240535. [Article in Portuguese] doi: 10.36660/abc.20240535 (PMC12017458; doi:10.36660/abc.20240535)
Supplement: Translation

# Translation: Fabry Disease and Its Different Phenotypes

**DOI:** 10.36660/abc.20240535i

**Authors:** Murillo Oliveira Antunes, Rafael Ruas Nastari, Edmundo Arteaga-Fernandez, Marcelle G. Henriques Lizandro, William Batah El-Feghaly, Guilherme José dos Santos Ferreira, Alan Silva Martins, Juliana Alzira Gonzales Oliveira Leguizamon, Vinicius Machado Correia, Vagner Madrini, Fábio Fernandes

**Affiliations:** 1 Brazil Instituto do Coração do Hospital das Clínicas da Faculdade de Medicina da Universidade de São Paulo, São Paulo, SP – Brazil; 2 Brazil Universidade São Francisco, Bragança Paulista, SP – Brazil

**Keywords:** Fabry Disease, Cardiomyopathies, Genetics

## Abstract

Fabry disease (FD) is an X-linked genetic condition caused by variants in the *GLA* gene causing enzyme α-galactosidase A deficiency and accumulation of globotriaosylceramide (Gb3) in tissues such as the heart, kidneys, and the nervous system. This study reports a case series of patients with FD, highlighting the phenotypic diversity of the disease, which can be confused with other cardiological conditions. When properly indicated, genetic evaluation, combined with biomarker dosage and α-galactosidase enzymatic activity, is key for an accurate diagnosis. Early diagnosis of FD is fundamental for initiating treatments that can slow disease progression and prevent serious complications, reinforcing the need for greater awareness about this condition among cardiologists.

## Introduction

FD is an X-linked lysosomal storage disorder caused by pathogenic variants in the *GLA* gene, which result in significantly reduced α-galactosidase A (α-GalA) enzyme activity. This leads to globotriaosylceramide (Gb3) accumulation in tissues such as the heart, kidneys, blood vessels, and the peripheral nervous system. Although incidence estimates vary, recent studies, including newborn screening and biobank analyses, suggest that the true prevalence may be underestimated, with an incidence of up to 1 in 5,732 for late-onset variants.^1^With the introduction of enzyme replacement therapy (ERT), early diagnosis has become crucial to slowing disease progression. This case series demonstrates the different phenotypes of FD, which can mimic other heart diseases, to facilitate the early recognition of the condition.

### Case 01 – Apical hypertrophic cardiomyopathy

A 29-year-old male patient, with no comorbidities or family history of heart disease, complained of tingling and burning in his legs as well as a spot in the abdominal region (angiokeratomas). He sought medical care due to atypical precordial pain ( Table 1 ). The electrocardiogram (ECG) showed a short PR interval and signs of left ventricular overload (LVO) with T wave inversion in V3-V6 ( Figure 1 ). The echocardiogram revealed asymmetric hypertrophy of the left ventricle (LV) in the apical region, with a maximum thickness of 17 mm and no obstruction in the LV outflow tract. The patient was initially diagnosed with non-obstructive hypertrophic cardiomyopathy (HCM), apical form. Cardiac magnetic resonance imaging (CMRI) showed circumferential hypertrophy in the middle and apical segments, with a thickness of 21 mm and multifocal myocardial late enhancement of a non-ischemic pattern ( Figure 1 ). Genetic investigation revealed a pathogenic variant in the *GLA* gene ( Table 2 ), and the alpha-galactosidase enzyme dosage showed a reduced value, confirming the diagnosis of FD. Family screening was performed and the same variant was found in the patient’s mother.

**Table 1:** – Clinical and Laboratory Characteristics

	Case #1	Case #2	Case #3	Case #4	Case #5	Case #6
Age, years	29	57	52	40	68	64
Gender	Male	Female	Female	Male	Female	Male
Phenotype	Apical HCM	Cardiac amyloidosis	Atrial fibrillation	Asymptomatic	(Heart Failure with Preserved Ejection Fraction) HFpEF	(Heart Failure with Reduced Ejection Fraction) HFrEF
Clinical Presentation	Cardiovascular symptoms – Chest pain	Hypohidrosis, acroparesthesia, and palpitations	Palpitations, acroparesthesia, and vertigo	Anhidrosis and heat intolerance	Cardiovascular symptoms – Dyspnea	Cardiovascular symptoms – Dyspnea
Short PR interval	Yes	*	Yes	Yes	Yes	*
LVEF, %	Preserved	Preserved	Preserved	Preserved	Preserved	Reduced
Maximum thickness, mm	21	15	21	12	19	17
α-galactosidase dosage	Reduced	Reduced	-	Reduced	-	Reduced
Lyso-gb3 dosage (range: up to 0.8 ng/ml)	3.1	15.71	-	71.7	23.1	-
Renal impairment	No	No	Yes	Yes	No	Yes

* Non-sinus heart rhythm. Source: The authors.

**Figure 1:** – Case 1: (A) Electrocardiogram with short PR interval and T wave inversion from V3 to V6. (B) CCMRI demonstrating LV apical thickening. Case 2: (C) Strain echocardiogram demonstrating LV involvement in anterior, lateral, and basal inferolateral regions. CCMRI with delayed enhancement in the LV lateral wall in the long axis (D) and short axis (E). Case 3: (F) Electrocardiogram with atrial fibrillation and right bundle branch block.

**Table 2:** – Genetic Tests

	Case #1	Case #2	Case #3	Case #4	Case #5	Case #6
Genetic Testing	Panel	Sanger	Sanger	Sanger	Sanger	Panel
Reference sequence	NM_000169.3	NM_000169.3	NM_00169.3	NM_00169.3	NM_00169.3	NM_00169.3
Chromosome Coordinate (GRCh37)	chrX-100653021	chrX-100662818	chrX-100656700	chrX-100655733-100655734	chrX-100655733-100655734	chrX: 100653930
c.DNA variant	c.1066C>T	c.73delG	c.467C>A	c.559_560del	c.559_560del	c.644A>G
Protein Variant	p.Arg356Trp	p.Asp25Thf*96	p.Ala156Asp	p.Met187ValfsTer6	p.Met187ValfsTer6	p.Asn215Ser
Zygosity	Hemizygosity	Heterozygosity	Heterozygosity	Hemizygosity	Heterozygosity	Hemizygosity
ACMG Classification	Pathogenic	Probably Pathogenic	Probably Pathogenic	Pathogenic	Pathogenic	Pathogenic

Source: The authors.

### Case 02 – Cardiac Amyloidosis Phenotype

A 57-year-old female patient, reported long-standing hearing loss and tinnitus, as well as hypohidrosis and pain in the lower limbs exacerbated by cold weather during childhood ( Table 1 ). Previously diagnosed with permanent atrial fibrillation (AF) and recurrent palpitations, she had undergone five unsuccessful ablation procedures. Her family history was poor for diseases of genetic origin. The echocardiogram showed moderate concentric LV hypertrophy, with a 114 g/m^2^mass index and preserved ejection fraction. The global longitudinal strain (GLS) of the LV was reduced, mainly in the basal segments of the anterior and lateral walls. CMRI revealed asymmetric LV hypertrophy, predominating in the inferolateral region, with a thickness of 15 mm, heterogeneous late enhancement of a non-ischemic pattern, and reduced myocardial native T1 (852 ms, reference ~1000 ms) ( Figure 1 ). Genetic testing identified a pathogenic variant in the *GLA* gene ( Table 2 ). The measured alpha-galactosidase enzyme activity was reduced, confirming the diagnosis of FD. The patient had two daughters and one son. The son tested positive for the same variant in family screening.

### Case 03 – Paroxysmal atrial fibrillation and renal failure

A 52-year-old female patient, on hemodialysis due to hypertensive chronic kidney disease was referred for cardiological evaluation after an episode of AF, reversed with amiodarone, during one of the hemodialysis sessions. She had always reported neuropathic pain in the extremities that worsened with cold and hot weather, as well as frequent episodes of vertigo ( Table 1 ). Her ECG showed sinus rhythm and short PR interval as well as right bundle branch block ( Figure 1 ). An echocardiogram was requested, which showed the interventricular septum was 21 mm thick and the lateral wall was 14 mm thick, with preserved ejection fraction and no obstruction in the LV outflow tract.

The patient reported a significant family history, including the death of her mother from chronic kidney disease, two brothers on hemodialysis, and a sister with non-dialysis chronic kidney disease. Given the family history associated with kidney and heart problems, a genetic analysis was performed which revealed a pathogenic variant in the *GLA* gene ( Table 2 ), also found in her brother, confirming the diagnosis of FD in the family.

### Case 04 – Asymptomatic with lateral wall hypertrophy

A 40-year-old male patient, reported heat intolerance and absence of sweating since childhood ( Table 1 ). He said that his mother had been diagnosed with cardiac arrhythmia. His ECG showed sinus rhythm with a short PR interval and signs of LVO. On echocardiogram, the left ventricular mass index was 142 g/m^2^, with septal thickness of 10 mm, lateral wall thickness of 12 mm, and hypertrophy of the papillary muscles ( Figure 2 ). Biochemical tests revealed impaired renal function and proteinuria of 1.55 g/24h. Given these findings, FD was suspected. The alpha-galactosidase enzyme activity dosage was reduced ( Table 1 ) and the genetic test (Sanger) revealed a pathogenic variant in the *GLA* gene ( Table 2 ), also identified in his mother.

**Figure 2:** – Case 4: (G) Echocardiographic images demonstrating thickening of the papillary muscle, increased global LV thickness, and strain technique with involvement of the basal segment of the inferior, lateral, and inferolateral walls. Case 5: (H) Echocardiographic image showing thickening of the LV septal and lateral wall. Case 6: (I) Echocardiogram reveals LV thickening as well as increased end-diastolic diameter. Optical microscopy images, stained with Masson’s trichrome, show areas of fibrosis (J) and hematoxylin eosin staining demonstrating extensive vacuolization of the cytoplasm of cardiomyocytes (K). (L) Coronal view of explanted heart with increased thickness of the entire myocardium.

### Case 05 – Heart failure with preserved ejection fraction

A 68-year-old female patient, complained of dyspnea on exertion, which significantly limited her daily activities. She reported episodes of atypical precordial pain, unrelated to the effort, ( Table 1 ) with no other complaints. When asked about her family history, she said that her father had died at the age of 45 from an undetermined cause. The ECG showed a short PR interval, intraventricular conduction disturbance, and diffuse alterations in ventricular repolarization. The echocardiogram showed concentric ventricular hypertrophy of the LV, as well as increased thickness of the interventricular septum and lateral wall of 19 mm and 18 mm, respectively. The calculated LV-indexed mass was 320 g/m^2^. The GLS was calculated and showed reduced values in the inferno-later-basal region ( Figure 2 ). A genetic panel for evaluation of HCM genocopies showed the presence of a pathogenic variant in the *GLA* gene, confirming FD ( Table 2 ). Two of the patient’s children underwent a family screening and the variant was identified in one of them.

### Case 06 – Heart failure with preserved ejection fraction

A 64-year-old male patient, with a history of HCM diagnosed 30 years ago, without other systemic symptoms ( Table 1 ), was admitted due to decompensated heart failure, profile C. Family history included a brother also diagnosed with HCM. Despite optimized treatment and cardiac resynchronization therapy performed three years earlier, the echocardiogram revealed an LV ejection fraction of 29% and ventricular hypertrophy with septal and lateral wall thicknesses of 17 mm ( Figure 2 ). Due to being refractory to inotropic withdrawal, the patient was considered to be in the terminal phase of the disease and underwent a successful heart transplant.

Histopathological analysis of the explanted specimen ( Figure 2 ) and a genetic panel ( Table 2 ) revealed a pathogenic variant in the *GLA* gene, with reduced levels of alpha-galactosidase enzyme activity. His brother was also sequenced for the same gene, and the same variant was found, of maternal origin.

## Discussion

FD is an X-linked genetic condition that manifests differently in men and women. There are two main phenotypes: the classic, more common in men, characterized by the total loss of α-GalA enzyme function, resulting in multisystemic symptoms; and the non-classical, with late-onset variants that leads to a slower progression of the disease, usually affecting only the heart or kidneys.^2 , 3^New studies have shown the tendency for loss-of-function variants to generate a phenotype with greatly reduced enzymatic activity and a classic clinical condition, while missense variants are associated with late-onset phenotypes.^4^In the series described, three patients had variants that resulted in an early stop codon, but only case 4 evolved as described in the literature.

Not only the genetic variant, but lyso-Gb3, the deacetylated form of Gb3, is a marker of FD strongly associated with its phenotype. Its value tends to be high in patients with the classic form and lower in the non-classical form.^5^

Male patients with late-onset FD have higher residual α-Gal A activity compared with classic FD, although still well below normal values.^1^In heterozygous women, α-Gal A activity may be normal or slightly deficient, often with asymptomatic clinical progression, which discourages serum measurement of this marker in this population, with the diagnosis being conditioned to genotype confirmation. In heterozygous women, the healthy X chromosome can be inactivated, with the one containing the variant being the one that predominates, generating severe symptoms in the classical clinical presentation of the disease. This genetic expression occurs randomly in each cell of the organism.^6^

Typical manifestations of FD, such as angiokeratomas and cornea verticillata, are less frequent, being more common in classical phenotypes. Adult patients with structural changes in the myocardium, progressive increase in myocardial thickening over time, conduction disorders, chronic kidney disease, and neurological symptoms, especially acroparesthesias and/or hypohidrosis, should be actively investigated for FD, as well as their family members.

The typical cardiac manifestation of FD is concentric left ventricular hypertrophy, common from the third decade of life onwards. This hypertrophy can be confused with other conditions, such as HCM and cardiac amyloidosis.^7^Studies indicate that FD is present in 0.5 to 3% of the cases diagnosed as HCM.^8^In the case series presented, two patients had the diagnosis of FD erroneously attributed to HCM, resulting in a delay in ERT and, in one case, the need for heart transplantation.

On the ECG, changes such as reduced PR interval and increased prevalence of AF are indicative of FD. Echocardiography is key for the diagnosis and monitoring of Fabry cardiomyopathy, revealing, among other findings, thickening of the ventricular wall and diastolic dysfunction.^2^

CMRI is also useful, highlighting fibrosis in the inferolateral basal segments and reduced T1 map values, which may be early indicators of FD.

Specific treatment consists of ERT to replace the missing enzyme, consequently preventing the accumulation of Gb3. In Brazil, we have alpha-galsidase and beta-galsidase, both administered intravenously every two weeks. More recently, a new therapy has been proposed, chaperones. These agents have emerged to increase the therapeutic toolkit in the treatment of FD. However, this class has only demonstrated efficacy in specific subtypes of pathogenic variants, requiring in vitro validation of the medication for the patient’s specific variant before its application. Chaperones act by maintaining the stability of the dysfunctional protein, assisting in its correct folding and, consequently, preserving its activity.^9^

Finally, a detailed clinical investigation, in addition to a genetic evaluation conducted by an experienced professional, is crucial for the diagnosis of FD. The ongoing training of cardiologists is essential to increase recognition of the disease and enable early interventions, which can slow disease progression and prevent serious complications, ensuring a better prognosis for patients.
